# The use of hydrolyzed formulas as a method of correction of feeding and clinical rehabilitation of infants with atopy signs

**DOI:** 10.25122/jml-2022-0254

**Published:** 2022-12

**Authors:** Volodymyr Volodymyrovych Bezruk, Oleksii Serhiiovych Godovanets, Oleksandr Hryhorovych Buriak, Nina Ivanivna Voytkevich, Olena Victorivna Makarova, Oksana Ivanivna Yurkiv, Michael Ivanovych Sheremet, Oleksandr Vyacheslavovych Bilookyi, Mykhailo Mykhailovich Hresko, Mariya Ivanivna Velia, Svyatoslava Vasylivna Yurniuk, Maryna Dmytrivna Hresko, Tetiana Sergiivna Bulyk, Larysa Vasylyvna Rynzhuk, Oleh Olehovich Maksymiv, Igor Dmytrovych Shkrobanets

**Affiliations:** 1 Department of Pediatrics, Neonatology and Perinatology Medicine, Bukovinian State Medical University, Chernivtsi, Ukraine; 2 Department of Foreign Languages, Bukovinian State Medical University, Chernivtsi, Ukraine; 3 Department of Patient Care and Higher Nursing Education, Bukovinian State Medical University, Chernivtsi, Ukraine; 4 Department of Surgery No.1, Bukovinian State Medical University, Chernivtsi, Ukraine; 5 Department of Pharmacy, Bukovinian State Medical University, Chernivtsi, Ukraine; 6 Department of Obstetrics and Gynecology, Bukovinian State Medical University, Chernivtsi, Ukraine; 7 Department of Prosthetic Dentistry, Bukovinian State Medical University, Chernivtsi, Ukraine; 8 Department of Medical and Organizational Management, National Academy of Medical Sciences of Ukraine, Kiev, Ukraine

**Keywords:** atopic dermatitis, children, formula feeding

## Abstract

Our study showed that in formula-fed babies, the use of mixture X (containing 2'-FL (2'-fucosyllactose) – a type of milk oligosaccharide, as well as carefully studied Bifidobacterium lactis, DHA, ARA, and nucleotides) as the main product for feeding could ensure children's body with all the necessary nutrients. Furthermore, it can minimize the progression of clinical signs of atopic dermatitis and reduce the use of drugs. In addition, there was an improvement in height and weight parameters, proportional development of the child (p<0.05), an increase in the number of erythrocytes (erythrocytes), hemoglobin (Hb), mean cell volume (MCV) (p<0.05), a decrease in the number of leukocytes (WBC) (p<0.05), and leveling of skin signs of atopic dermatitis (AD) (according to SCORAD a decrease from 32.8±5.5 to 16.1±2.2, p<0.05). Based on these results, it is possible to recommend using mixture (X) with a preventive and rational purpose as a product of artificial feeding of children with a hereditary predisposition to allergies.

## INTRODUCTION

Allergy is a common condition that occurs when the immune system overreacts to certain substances such as pollen, dust, or certain foods. Atopic dermatitis (AD) is one of the most common forms of allergy, and although it can be diagnosed at any age, most often, its debut occurs during the first years of life [1–5].

One of the main rehabilitation approaches for children with AD is elimination diet therapy. In the case of a rational diet, it is important to remember that breast milk is the most optimal, specific hypoallergenic product for infants. It is essential to choose a therapeutic diet appropriate for a child's age and nutritional needs, particularly when atopic signs are present, and formula feeding can be a suitable option for infants during the first six months of life. To provide optimal feeding in such cases, special attention should be paid to the selection of formula which could provide the whole spectrum of essential nutrients and prevent allergy [6–14].

This study aimed to evaluate the effectiveness of the formula (Х) as an optimized partially hydrolyzed protein complex containing 2'-FL (2'-Fucosyllactose) – a type of milk oligosaccharide and extensively studied Bifidobacterium lactis, DHA, ARA, and nucleotides in managing atopic signs and as a mixture of choice concerning rational formula or mixed feeding of infants.

## MATERIAL AND METHODS

This analytical study used randomized sampling and included 20 infants with atopic dermatitis. Infants were fed on formula and mixed feeding with standard formulas. The children were between 1 and 7 months old at the time of the first examination. Transfer to formula feeding (Х) was made according to the 3^rd^ and 7^th^-day schemes. After that, a further amount of formula was regulated according to the manufacturer's instructions and the child's needs. The general period of formula feeding without administration of any pharmacological agents before repeated clinical and laboratory examinations was 8 weeks. During this period, new types of correction or food were not introduced.

The research included clinical examination (assessment of children's health and AD severity using SCORAD) [15, 16], somatometry (assessment of physical development and proportional development of children), social-metric examination (determination of external and internal factors of micro social environment employing a comprehensive anamnesis and questionnaire of parents), and laboratory tests (hemogram with the automatic blood analyzer ADVIA^®^, Bayer). In addition, the anthropometric parameters of children were analyzed using standard regional indices of physical development [17]. Finally, the data obtained were processed using standard parametric and nonparametric statistics [18, 19].

## RESULTS

Out of the 20 children examined, 16 infants (80.0%) received formula feeding, and 4 (20.0%) mixed feeding. The data of the social-metric study showed that 1 child (5.0%) had atopic signs since birth, 8 children (40.0%) had food sensitization to animal milk, 15 (75.0%) – allergy to standard milk formulas, both adapted and low adapted, 5 children (25.0%) had fruit allergy, and 2 infants (10.0%) – allergy to medicines and insect bites. Aggravated family history of allergies was registered in 5 children (25.0%).

**Figure 1:**
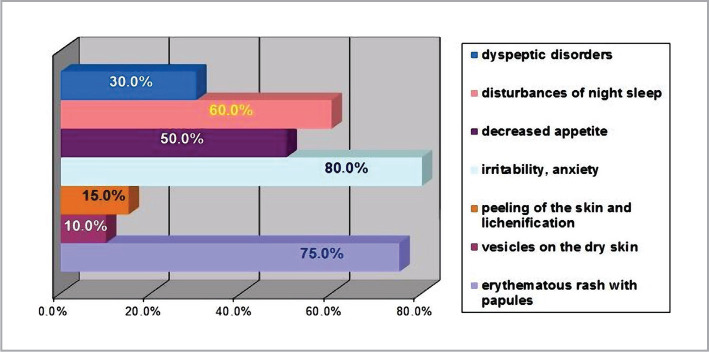
Clinical signs of AD in the children examined.

Clinical examination of children revealed the following skin signs: erythematous rash with papules, vesicles on the dry skin, peeling, and lichenification. General signs included behavioral disturbances (irritability, anxiety), changes in eating behavior (decreased appetite), disturbance in night sleep, and dyspeptic disorders (Figure 1).

Tolerance to the formula (X) was analyzed when children were transferred to formula feeding (Table 1).

**Table 1:** Tolerance indices to the formula (X).

Indicator of intolerance	3 days introduction	7 days introduction
**Stool disorders**	2 infants (10.0%)	2 infants (10.0%)
**Short-term flatulence**	3 infants (15.0%)	1 child (5.0%)
**Regurgitation**	3 infants (15.0%)	2 infants (10.0%)
**Thirst, behavioral disturbances**	0%	0%

The assessment of the anthropometric parameters revealed adequate metabolism of the formula and its positive effect on children's growth and weight parameters and proportional development (Table 2).

**Table 2:** Dynamics of children's anthropometric parameters on formula feeding (X).

Parameters	First examination		Second examination		McNemar Test
	M±m	% Children in the middle percentile corridor (25–75%)	M±m	% Children in the middle percentile corridor (25–75%)	p
**Body weight (kg)**	7.14±1.52	66.67	8.16±1.46	88.89	p=0.016
**Body length (cm)**	64.01±5.22	77.78	68.01±4.99	83.34	p=0.015

In what concerns laboratory findings, there was an increased amount of red blood cells (RBC), hemoglobin (Hb), mean cell volume (MCV), and a decreased amount of white blood cells (WBC) (Table 3).

**Table 3:** Hemogram results of children on formula feeding (X).

Indices	First examination	Second examination
**RBC, t/l**	3.78±0.61	4.39±0.61*
**Hb, g/L**	97.71±15.95	117.26±13.75*
**MCV, fl**	78.82±6.58	82.76±4.35*
**WBC, g/L**	10.16±1.95	8.90±1.76*

RBC – red blood count; Hb – hemoglobin; MCV – mean corpuscular volume; WBC – white blood cell; * – probability of difference with the first examination (p<0.05).

**Figure 2:**
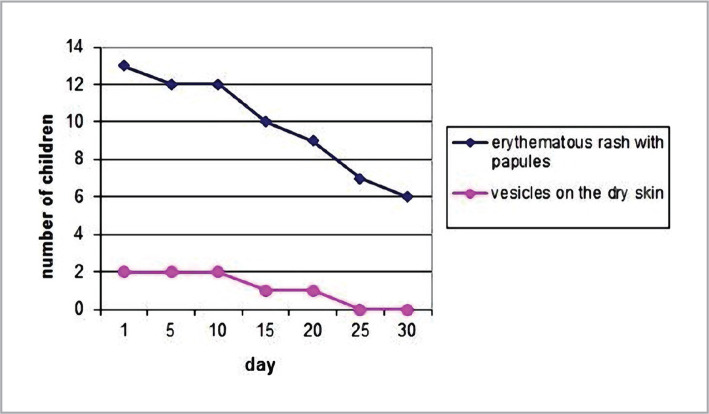
Dynamics of skin changes in children with AD.

**Figure 3:**
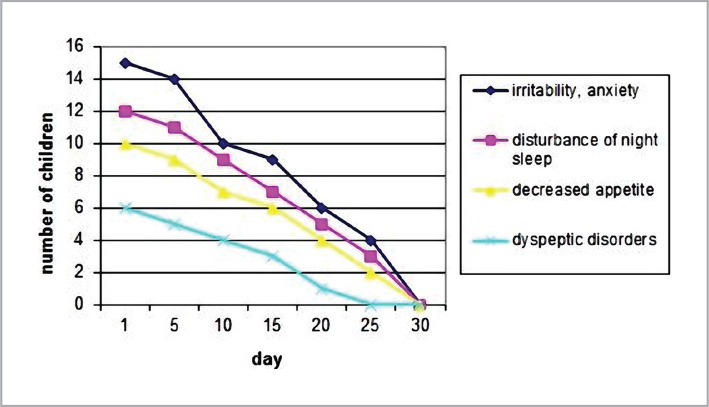
Dynamics of extracutaneous manifestations of AD.

The dynamics of skin signs (according to SCORAD) were analyzed on the 5^th^, 10^th^, 15^th^, 20^th^, 25^th^, and 30^th^ days. After 3–4 weeks of formula feeding, the skin signs of AD were leveled (decrease according to SCORAD from 32.8±5.5 to 16.1±2.2, p<0.05), and extracutaneous manifestations of AD regarding the nervous system and digestive tract reduced (Figures 2 and 3).

## DISCUSSION

The role of hydrolyzed formulas in the prevention and complex treatment of atopic dermatitis and other infant allergic diseases has been actively discussed since 2008 when the American Academy of Pediatrics Committee on Nutrition (AAP) published a report on the influence of infant atopic disease development and children's early nutritional correction, as well as the terms of introducing formula feeding and the use of hydrolyzed one in particular [20].

Further studies provide many controversial results that hydrolyzed formula can reduce the risk of infants' allergic illnesses and prevent children from atopic disease. Today, the issue of whether hydrolyzed formula prevents infants who are formula fed from atopy or other allergic diseases is still being discussed. Also, the link between using hydrolyzed formulas when feeding babies with high risks of allergic disease development and its decrease is questioned, too [3, 21–24].

At the same time, we can report that we have used hydrolyzed formulas in high-risk infants to improve atopy symptoms and ensure optimal rational formula or mixed feeding of infants after birth. Nevertheless, the results of current studies show the need for further research on the role of hydrolyzed formulas in infant nutrition and their preventive influence on atopy development.

## CONCLUSIONS

The positive clinical results of the research suggest that formula (Х) as an optimized, partially hydrolyzed protein complex containing 2'-FL (2'-Fucosyllactose) – a type of milk oligosaccharide and extensively studied Bifidobacterium lactis, DHA, ARA, and nucleotides, in infants with atopy debut who are on formula or mixed feeding, eliminates the signs of atopy and prevents the progression of AD. Furthermore, it improved the growth-weight parameters and hematological indices, promoting proportional physical development and minimizing the administration of pharmacological agents in treating AD.
